# Kynurenine pathway metabolites in migraine

**DOI:** 10.1186/1129-2377-16-S1-A1

**Published:** 2015-09-28

**Authors:** Ferdinando Nicoletti

**Affiliations:** Dipartimento di Fisiologia Umana e Farmacologia “V. Erspamer”, Università Sapienza di Roma, Rome, Italy

**Keywords:** Migraine, Metabotropic Glutamate Receptor, Quinolinic Acid, Cortical Spreading Depression, Kynurenic Acid

Kynurenine pathway (KP), the quantitatively main branch of tryptophan metabolism, has long been considered a source of nicotinamide adenine dinucleotide, although several of its products, the so-called kynurenines, are endowed with the capacity to activate glutamate receptors, thus potentially influencing a large group of functions in the central nervous system (CNS). In fact, Kynurenic Acid and Quinolinic Acid are able to interact with ionotropic glutamate receptors and Cinnabarinic Acid has been reported as an orthosteric agonist of metabotropic glutamate receptors (mGlu4), and Xanthurenic Acid has been recently demonstrated to be a putative agonist of metabotropic glutamate receptors 2/3 (mGlu2/3). Moreover, 3-HK and 3-HANA have mainly been studied, since they have been shown to induce neurotoxic effects by increasing oxidative stress and the production of free radicals or through excitotoxicity. Migraine has a complex pathophysiology in which both central and peripheral components of the trigeminal pain pathway play a central role. The trigemino-vascular activation during the attack has largely been described, and recently the brainstem nuclei, called “migraine generators”, have been reported to be involved in migraine. Moreover, a series of destabilizing events within the brain trigger a cortical spreading depression (CSD), responsible for the aura phenomena and for trigeminal activation. The role of glutamate is heavily supported both in the trigemino-vascular as well as in brainstem nuclei activation, and furthermore in the CSD initiation and propagation. Some of the KP metabolites able to interact both with ionotropic and metabotropic glutamate receptors might be involved in migraine pathophysiology. Despite the large number of studies conducted on migraine etiopathology, the KP has only been recently linked to this disease. Nonetheless, some evidence suggests an intriguing role for some kynurenines, and an exploratory study on the serum kynurenine levels has been helpful to better understand possible alterations of the kynurenine pathway in patients suffering from migraine.

